# Accommodative Response in Patients with Central Field Loss: A Matched Case-Control Study

**DOI:** 10.3390/vision5030035

**Published:** 2021-07-09

**Authors:** Ali Mazyed Alsaqr, Hisham AlShareef, Faisal Alhajri, Ali Abusharha, Raied Fagehi, Ahmad Alharbi, Saud Alanazi

**Affiliations:** Department of Optometry, College of Applied Medical Sciences, King Saud University, Riyadh 11433, Saudi Arabia; ioi7@live.com (H.A.); h.alshareef23@gmail.com (F.A.); aabusharha@KSU.EDU.SA (A.A.); rfagehi@KSU.EDU.SA (R.F.); ahmaalharbi@KSU.EDU.SA (A.A.); saaalanazi@KSU.EDU.SA (S.A.)

**Keywords:** accommodation, macular degeneration, pediatrics, visual impairment, accommodation lead, accommodation lag

## Abstract

**Purpose**: This study was conducted to evaluate the accommodative response in young participants with visual impairment in comparison with visually normal participants. **Methods**: Fifteen participants with confirmed visual impairment and 30 visually normal participants aged 12–15 years were recruited. Accommodative response was measured using autorefractor (Grand Seiko WV500) at distances of accommodative demand of 33, 25, and 20 cm. The targets were one-line-above participant threshold acuity. The participants’ accommodative responses were compared between both groups after calibration for refractive errors and the vertex distance of the glasses. Visual acuity and refractive status were also assessed. **Results**: The age was not significantly different between both participant groups. The visual acuity of visually impaired patients was 6/30 to 6/240, and that of visually normal participants was 6/7.5 or better. Ten of the visually impaired patients and 29 of visually normal participants were myopic. In total, 61–73% of visually impaired patients showed an accommodative lead. Five subtypes of accommodative response were observed. In general, the accommodative inaccuracy increased with increasing accommodative demand. However, the visually normal participants largely exhibited an accommodative lag. A mild-to-moderate relationship was observed between visual acuity and accommodative response (*r* = 0.3–0.5, *p* < 0.05). **Conclusion**: Accommodative response in young visually impaired patients can be variable and on an individual basis. Low vision specialists should anticipate accommodative response outside the normal range. Therefore, we shall consider evaluating each patient’s accommodative response before prescribing any near addition lenses. Accommodation inaccuracy is often more complex than predicted due to increased depth of focus caused by reduced visual acuity.

## 1. Introduction

The change occurring in the optical system to maintain a clear image of an object as its distance varies is termed accommodation [1]. Abnormalities in accommodation response could result in binocular vision problems and consequently reduce the efficiency of the visual system [2]. The accommodative function varies considerably from one individual to another [3,4].

Accommodative response (AR) takes place when the eye changes its fixation from a distant point in space to a nearby object [3,5]. The difference between the accommodation demand and AR can be greater than the accommodative demand (accommodative lead), equal to the accommodative demand, or less than the accommodative demand (accommodative lag) [6,7]. The AR is an important parameter in clinical diagnosis of non-strabismic disorders of binocular vision and is widely applied in the clinical setting [8,9,10,11,12,13,14]. The AR changes rapidly and continuously, and these microfluctuations range approximately from 0.20 to 0.50 D [15]. Subjective (MEM retinoscopy, Nott dynamic retinoscopy, dynamic cross-cylinder, and near red-green duochrome test) and objective methods (autorefractor) were proposed for measuring the AR at certain accommodative demands. However, studies have suggested that these methods do not provide consistent or interchangeable results [16,17].

Several factors can affect AR, including visual acuity (VA) [18], age [19,20], refractive status of the eye [21,22], near-point target distance [23], heterophoria [24,25], accommodative insufficiency or accommodative excess [26], exophoria or convergence excess [27], visual symptoms [28,29], and illumination level and cone activity [30,31].

Patients with visual impairment (VI) (e.g., age related macular degeneration, diabetic retinopathy, glaucoma, and corneal opacity) prominently have reduced VA that could lead to unreliable AR [32,33]. Some of these patients do not visit the clinic with complaints that are suggestive of accommodative difficulties [34]. One explanation is that these patients could depend on cues other than or in addition to defocus blur to effectively drive accommodation [34].

Previously, some cross-sectional studies investigated AR in patients with VI at certain points of age but reported conflicting results [34,35,36]. White and Wick investigated the AR of patients with juvenile macular degenerations [34], with SRI servo-controlled punctuation infrared optometer and ranging in age from 19 to 34 years. They detected poor AR, where their subjects tended to have accommodative lead at near-viewing distances ranging from 0 to 5 D. Leat and Mohr evaluated patients with low vision in the age range of 5–31 years with variable conditions, including refractive amblyopia, measured by dynamic retinoscopy, at accommodative demands of 4, 6, 8, and 10 D [35]. They found an accommodative lag, a contrary result from White and Wick study. They also reported that the lag of accommodative responses increased with increasing target demands. In addition, Heyman evaluated AR in children aged 5–15 years with congenital macular disorders and moderate-to-severe VI using the Grand Seiko WV500 autorefractor at accommodative demands of 3–5 D [36]. They reported different results from the two previous studies. Where the AR of children with VI exhibited larger variability, and the children had deficits in their AR, with 50% of them having accommodative lag and 20% having accommodative lead.

The present study was conducted to investigate the AR of young adults with VI in the age range of 12–15 years, an issue that has not been fully explored previously. The potential differences in AR between young adults with VI and healthy subjects were also evaluated. Understanding the accuracy of accommodation in young patients with VI is an important factor when prescribing optical devices [36]. Unlike previous research, this study focused on only young patients with VI and used a completely objective accommodation measurement using an open-field autorefractor (Grand Seiko WV500, Grand-Seiko Company, Fukijama, Japan). To the best of our knowledge, there are no previous studies that have investigated the AR of young patients with VI compared with young healthy participants (HPs).

## 2. Methods

The study was conducted over a period of 8 months. This study recruited 15 young participants with VI and 30 HPs without any other ophthalmic pathology (aged 12–15 years). Past history of the participants with VI was reviewed to determine the diagnosis and VA for study eligibility. Macular degeneration was identified as the cause of reduced VA in all participants with VI. VA was evaluated using the Early Treatment Diabetic Retinopathy Study (ETDRS) chart. Noncycloplegic refraction was used to confirm the participants’ habitual distance correction.

The Grand Seiko WV500 (Grand-Seiko Company, Fukijama, Japan) open-field autorefractor was used to measure AR at demand distances of 50 cm (2 D stimulus), 33 cm (3 D stimulus), and 20 cm (5 D stimulus) using a vertical high-contrast grating pattern that is one-line above each patient’s threshold VA and a near-point rod and custom made near-point target holder. The holder allows the near-point target to be moved closer to the eye under the instrument’s beam-splitter housing. At each test distance, the target size was maintained at one-line above the participant’s threshold. The room illumination was full to allow the participants to view the near chart clearly. For testing, the participants were seated at the instrument with their head stabilized in the chin rest and forehead strap. The test was conducted monocularly, during which the other eye was covered with the instrument’s occluder. The autorefractor measurements were repeated three times for each distance, and the average of the three readings (spherical equivalent) was recorded as the representative reading. The entire testing was conducted through the subject’s best distance refraction. This study used the formulas suggested by Atchison and Varnas (2017) to calculate the accommodative stimulus, accommodative response, and accommodative lag/lead for each participant at the three accommodative demands [37]. The calculation steps of the three formulas are

$$\mathrm{AS}=\frac{-L}{\left(1-d.SR\right)\left[1-d\left(L+SR\right)\right]}$$

where AS represents accommodative stimulus; *L*, object vergence; *d*, vertex distance; and *SR*, spectacle refraction.

$$\mathrm{AR}=\frac{-R}{\left(1-d.SR\right)\left[1-d\left(R+SR\right)\right]}$$

where AR represents accommodative response; *R*, autorefractor reading; *d*, vertex distance; and *SR*, spectacle refraction.

AL=AS−AR

Giving that accommodative lag (*AL*) if positive and accommodative lead if negative.

The IBM SPSS Statistics for Windows, version 22 (IBM Corp., Armonk, NY, USA), were used for data analysis. Data were not normally distributed as assessed by the Kolmogorov–Smirnov test (*p* < 0.050). Median ± interquartile ranges were used to report the data. The Mann–Whitney test was used to examine the difference in AR between participants with VI and HPs. Differences were considered to be statistically significant when the *p* value was <0.05.

The AR was measured monocularly during the phase of data collection. The Spearman test was conducted to investigate the relationship between the outcomes of the right and left eyes in both study groups. The test showed a very strong significant relationship between both eyes in both groups (*r* ≥ 0.90, *p* < 0.0001). Therefore, the AR of one eye from each participant in both groups was randomly selected, and further analysis was conducted. This was done to avoid any eventual correlation existing between the right and left eyes of a single patient [38,39].

## 3. Results

**Figure 1:**
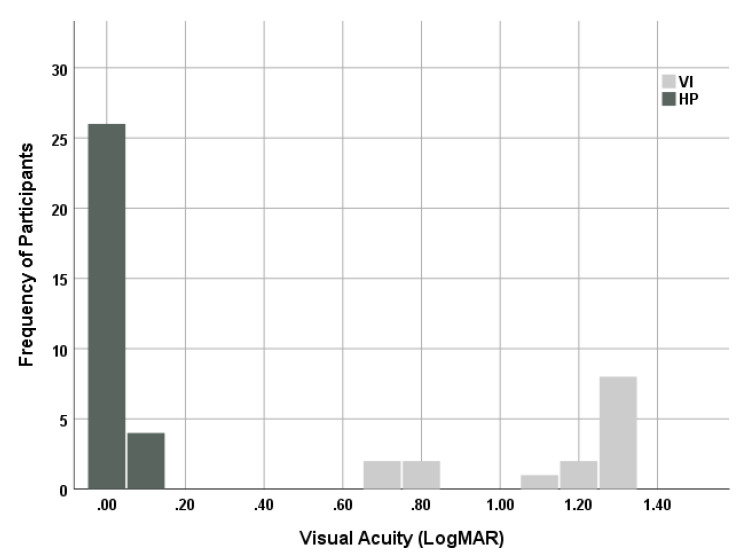
Illustration of the distribution of visual acuity of both visual impairment group (VI) and participants with healthy vision (HP).

The age of participants in both study groups was similar (Table 1). The VA of participants with VI with the best optical correction of 1.30 ± 0.70 logMAR (6/30 to 6/240) and that of HPs with best optical correction were within the normal limit of VA (Table 1, Figure 1). A statistical difference was observed between both groups in the VA scores (Table 1). The calculated accommodation stimulus (AS) at the three near distances showed that the actual AS at 50, 33, and 20 cm were 1.97 ± 0.06 D, 2.96 ± 0.1 D, and 4.9 ± 0.14 D, respectively.

In the VI group, 10 patients (67%) were myopic (−0.25 to −6.50 D), 8 patients had myopia less than −2.50 D, 4 patients (27%) had hyperopia (0.50–6.00 D), and a patient had emmetropia (Table 1). In the HP group, 29 participants (97%) were myopic (−0.50 to −2.50 D), 16 participants had myopia <1.00 D, and 1 participant had hyperopia (1.00 D) (Table 1). The cylindrical component in the HP group was <1.00 DC, whereas 13 patients with VI had a cylindrical power of less than −2.00 DC.

**Figure 2:**
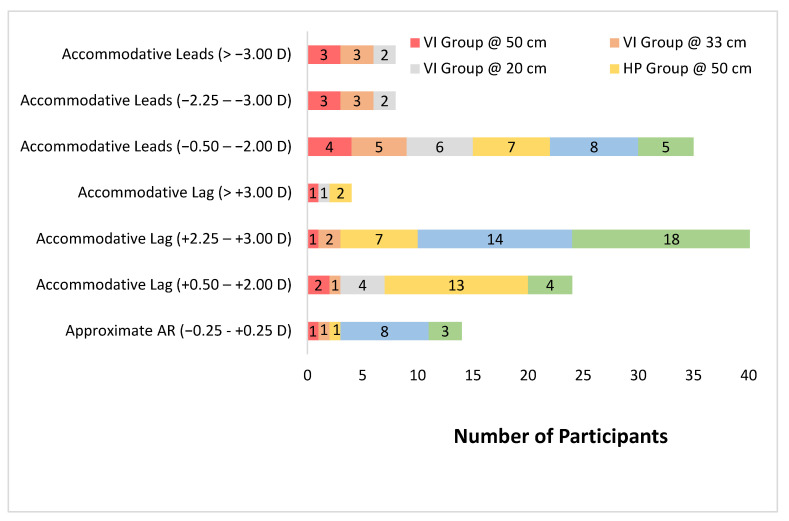
Frequencies of the accommodative responses of control participants and visual impairments participants under the three conditions: 50, 33 and 20 CM.

In terms of AR, the majority of patients with VI had accommodative lead (Figure 2). In detail, at demand distances of 50, 33, and 20 cm, there were 67% (10 of 15), 73% (11 of 15), and 67% (10 of 15) of participants with accommodative lead, respectively. The accommodative lag was 25% in this group (Figure 2). In contrast, the majority of participants in the HP group had accommodative lag (Figure 2). Specifically, at demand distances of 50, 33, and 20 cm, there were 66% (20 participants), 48% (14 participants), and 73% (22 participants) of participants with accommodative lag, respectively. The accommodative lead ranged from 16 to 25% in the HP group (Figure 2). Overall, a significant difference was observed in AR between the VI and HP groups (Table 1).

The five subtypes of AR classification that were defined and used by Heyman were also applied in this study for comparison purposes [36]. The five subtypes were negative slope lag, fixed lag, fixed accurate, positive slope lag, and fixed lead. In detail, a negative slope lag shows increasing lags as the demand increases; fixed lag indicates a tendency to under-accommodate a steady amount regardless of the accommodative demand, while fixed accurate range represents an AR that is similar to approximate normal AR. Additionally, positive slope lag refers to marked lag of accommodation at first and then much more accurate AR as the demand increases. Finally a fixed lead indicates an over accommodation that decreases as the demand increases [36].

**Figure 3:**
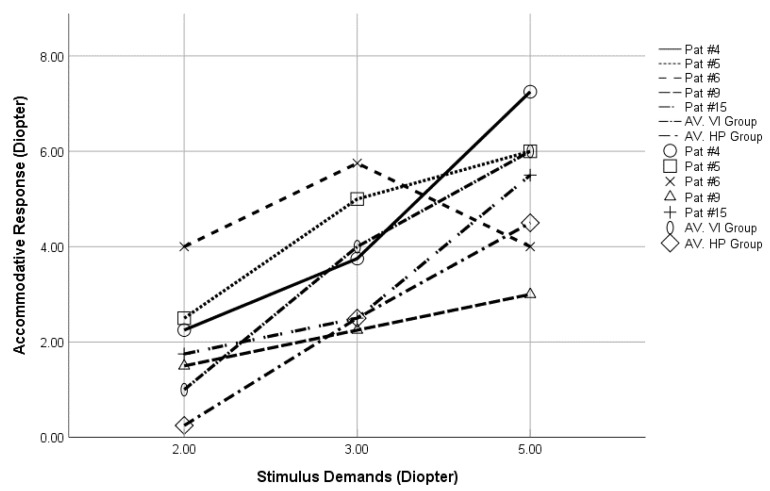
A sample of accommodative responses observed in this study, those are patients’ numbers 4, 5, 6, 9, and 15 that demonstrate the five subtypes founds at the three accommodative demands 2, 3, and 5 Diopters. The average accommodative responses for visual impairment group (AV.VI group) and for participants with healthy vision (AV.HP group) was also illustrated.

In this study, the patients’ AR fell into three subtypes of those subtypes and an additional two subtypes were observed (Figure 3 and Table 2). In detail, two participants were observed with fixed accurate subtypes. Three participants had negative slope lag subtypes and four participants with fixed lead subtype. In comparison with the study of Heyman, none of our study participants had a “positive slope lag” or “fixed lag” subtypes [36]. We also observed the “negative slope lead” subtype (three participants) that showed increasing leads with the increase in demand. Finally, the “variable slope” subtype (three participants) demonstrated accommodative leads at 2–3 D demands and accommodative lag at 5 D.

In the VI group, the relationship among participants’ age, VA, and refractive errors with AR under the three conditions demonstrated that VA was the major determinant of AR at the three demands (r = −0.41, *p* = 0.003, r = −0.54, *p* < 0.001, r = −0.1, *p* = 0.5, respectively). However, the strength of this relation does not fully explain the AR and would suggest that the AR is more complex than VA alone.

## 4. Discussion

The findings of this study supported earlier findings that patients with VI accommodate less accurately than a normally-sighted person. However, our study was different in several aspects. It is a case-control study, highlighting the differences between AR in participants with VI and HPs. Our study also targeted a cross-sectional group of age (i.e., 12–15 years) that has not been investigated sufficiently. Specifically, White and Wick recruited participants in the age range of 19–34 years; in the study of Leat and Mohr, only two participants were recruited (of the 21 participants aged 5–31 years) in the same age group of our study; and Heyman recruited five participants in the age range of 12–15 years out of the 10 patients recruited.

Leat and Gargon recruited a group of normal children aged 4–15 years, and it was found that the older age group demonstrated underaccommodation that increased with accommodative demand [40]. It has also been suggested that a small lag of accommodation would be present in healthy subjects under a normal situation with a steady-state near-point stimulus [41]. Heyman suggested that the AR of normal subjects was reasonably accurate, with errors for individual subjects ranging from 0.20 to 0.84 D [34]. However, our study showed that patients with VI are distinct from those with normal VA, a finding that is also in agreement with previous studies [34,35,36]. A previous research reported no significant difference in AR between subjects with emmetropia and those with myopia [18]. Other authors have suggested that subjects with myopia accommodate less than those with emmetropia or hyperopia when viewing an increasingly near target [42,43]. Gwiazda et al. also suggested that progressing myopes have a tendency to demonstrate a larger lag in accommodation than do emmetropes subjects [44]. In our study, there was an imbalance in the number of myopes in each group, which may have a potential confounding effects on the differences in AR that we found between the VI and HP groups. Finally, the finding of the HP group AR supports the previous evidences that myopes have a tendency to have an accommodation lag.

Our study findings are in agreement with three of the five subtypes suggested by Heyman and also found two additional subtypes. This result indicates that AR could vary markedly between individuals. Consistent with the study of White and Wick, this result also emphasizes that visually impaired patients are generally overaccommodating [34]. These results were different from those reported by Leat and Mohr and Heyman, which may be attributed to the differences in the age and conditions of the recruited participants (e.g., Leat and Mohr recruited patients with refractive amblyopia). Leat and Mohr suggested that their finding of the lag in AR with increasing demands might be a result of fatigue effect affecting the higher demands [35]. Furthermore, the variation in AR observed in the VI group in our study could be partially attributed to sensory abnormalities (i.e., reduced VA, reduced contrast sensitivity, and increase in the depth of focus) [34]. The decline in those parameters could lead to limiting the participant’s ability to detect the need for accommodative change and subsequently may cause the AR inaccuracy [34]. Based on the results of our study, low vision specialists could recommend evaluation of AR in patients with low vision as patients with accommodation lag might require plus lenses for near works as suggested previously by Heyman [36]. It should also be noted that patients with VI may have variable response based on their age, experience, adaptation, and the required daily activities.

In the present study, AR was significantly different between the VI and HP groups. This result was similar to that suggested in previous reports. Patients with macular pathology demonstrated less accurate AR [34,36]. Lastly, Leat and Mohr reported that their study participants’ AR was outside the 95% range of normal [35].

The AR of the visually normal population to a specific near demand was investigated previously [16,17,18,45]. Those studies demonstrated that accommodative lag was the primary finding, and it was up to 0.75 D. D Our findings on HP outcomes were generally consistent with those findings. The generally low response (underaccommodation) of our study participants in the HP group could be partially attributed to their inexperience [34].

The AR and VA showed a mild-to-moderate relationship (*r* = 0.3–0.5). Previous studies have suggested that reduced VA is not a sufficient explanation for the reduced accommodative accuracy [34,35,36]. An earlier study also reported that reduced accommodation was not predicted by age, VA, presence of nystagmus, refractive error, or the time of onset of the disorder [35]. The authors of that study concluded that accommodation responses are also more complex than predicted by the increased depth of focus due to poor VA [35]. Specifically, Grean et al. suggested a formula in which the worse the VA recorded, the larger the depth of focus [46]. Legge et al. also provided evidence of the inverse relationship between the VA and the depth of focus [47]. The nature of this relationship can be accounted for by the fact that when perceived fine detail is reduced due to decline in VA, the perception of blur will take place. Therefore, the poorer the VA, the bigger the just-detectable blur circle on the retina that will be appreciated for a defocused point in the object. Subsequently, the larger just-detectable blur circle would result in a larger depth of focus [2]. These suggestions of the AR complex nature are in agreement with our findings. Other factors may involve the outcome of AR in patients with VI, including fixation stability, eye movement, stress, fatigue, adaptation to near working distance, and other factors that may influence the overall AR outcome.

The results of this study could indicate that in the clinic, young patients with reduced VA have variable AR. Patients with VI generally hold the reading material at distances less than the habitual 40 cm. Previous research suggests that under our binocular condition, patients with macular degeneration should be able to accommodate fairly accurately [34]. It has also been suggested that young patients with macular degeneration infrequently complain about their near vision. However, due to the constant closer viewing distance, their accommodative demand will be fairly higher than the normal accommodative demand (2.50 D). Eventually, these patients would experience the effects of presbyopia at younger ages and require a reading lens of higher power than that required by a normally sighted person [34]. In addition, some pediatric patients with VI are frequently prescribed near addition lenses to meet the high accommodative demand [48]. Therefore, clinicians should consider this aspect when dealing with patients with reduced VA. The AR should be measured in any patients with VI using any clinical tool available before prescribing any near reading optical aid. Providing the appropriate near-reading optical aids in this population is important for achieving optimum reading outcomes [36].

There were some limitations in the present study. The first limitation was the small sample size, although it is challenging to recruit a large sample from this population as reported by previous studies [34,35,41]. However, this limitation did not allow for the analysis of AR in relation to specific disease conditions. The second limitation was the cross-sectional design of our study, due to which the long-term effect and/or the training effect could not be concluded. Future research on examining the AR during follow-up visits to determine the training factor might provide a valuable insight into AR in this population. Another limitation was that the Grand Seiko WV500 autorefractor was able to measure the variability of the AR (SD) during near visual task. This criterion was of importance as previous studies suggested that the AR of children with VI exhibited larger variability. It is suggested that future studies involve this outcome in investigating AR in patients with visual impairment. Finally, although the open-field autorefractor provides an objective measurement of AR, it has the physical limitation of providing measurements up to 5D. Some children with VI adopt a closer working distance. It is recommended that clinicians should use their available clinical resources to evaluate AR before prescribing near-vision corrections. A.A. (Ahmad Alharbi). 

## Figures and Tables

**Table 1:** Summary of the participants’ characteristics and error of accommodation under the three conditions: 50, 33, and 20 CM in both participants groups.

Variable	VI Group Median ± IQ (Range)	HP Group Median ± IQ (Range)	Mann Whitney Test
Age (Years)	14 ± 1.00 (12–15)	14 ± 2.00 (12–15)	Z = −1.21, *p* = 0.23
VA LogMAR	1.3 ± 0.7 (0.70–1.8)	0.00 ± 0.00 (0.00–0.1)	Z = −6.044, *p* < 0.0001 *
Sph.Eq	−3.25 ± 4.00 (−6.50–+6.00)	−1.25 ± 1.00 (−2.50–+1.00)	Z = −1.455, *p* = 0.14
AR @ 50 cm	1.14 ± 4.86 (−13.75–+3.00)	2.45 ± 1.50 (−1.50–+3.00)	Z = −2.25, *p* = 0.025 *
AR @ 33 cm	−1.00 ± 2.90 (−14–+2.00)	0.25 ± 1.25 (−1.25–+1.50)	Z = −2.9, *p* = 0.003 *
AR @ 20 cm	−1.00 ± 2.75 (−16–+4.00)	0.50 ± 1.50 (−0.50–+2.25)	Z = −2.2, *p* = 0.03 *

VA; visual acuity, Sph.Eq; spherical equivalent; AR, accommodative response, *; indicate significant level *p* < 0.05.

**Table 2:** The age, visual acuity, and associated accommodative response subtype for each participant in the visual impairment group.

Patient	Age	VA (LogMAR)	Error of Accommodation
1	14	1.3	negative slope lead
2	12	1.3	negative slope lag
3	15	1.5	variable slope
4	14	1.3	variable slope
5	14	1.8	negative slope lead
6	12	1.3	fixed lead
7	12	1.3	variable slope
8	15	1.5	fixed lead
9	15	1.3	negative slope lead
10	15	1.3	negative slope lag
11	15	0.8	negative slope lag
12	14	1.8	fixed lead
13	15	0.7	fixed accurate
14	15	0.8	fixed lead
15	14	0.7	fixed accurate

## Data Availability

The data presented in this study are available on request from the corresponding author.
